# Assessment of Rural-Urban Differences in Postacute Care Utilization and Outcomes Among Older US Adults

**DOI:** 10.1001/jamanetworkopen.2019.18738

**Published:** 2020-01-08

**Authors:** Cyrus M. Kosar, Lacey Loomer, Nasim B. Ferdows, Amal N. Trivedi, Orestis A. Panagiotou, Momotazur Rahman

**Affiliations:** 1 Department of Health Services, Policy and Practice, Brown University School of Public Health, Providence, Rhode Island; 2 Center for Gerontology and Healthcare Research, Brown University, Providence, Rhode Island; 3 Edward R. Roybal Institute on Aging, University of Southern California, Los Angeles

## Abstract

**Importance:**

Although people living in rural areas of the United States are disproportionately older and more likely to die of conditions that require postacute care than those living in urban areas, rural-urban differences in postacute care utilization and outcomes have been understudied.

**Objective:**

To describe rural-urban differences in postacute care utilization and postdischarge outcomes.

**Design, Setting, and Participants:**

This retrospective cohort study used data from Medicare beneficiaries 66 years and older admitted to 4738 US acute care hospitals for stroke, hip fracture, chronic obstructive pulmonary disease, congestive heart failure, or pneumonia between January 1, 2011, and September 30, 2015. Participants were tracked for 180 days after discharge. Data analyses were conducted between October 1, 2018, and May 30, 2019.

**Exposures:**

County of residence was classified as urban or rural using the US Department of Agriculture Rural-Urban Continuum Codes. Rural counties were divided into those adjacent and not adjacent to urban counties.

**Main Outcomes and Measures:**

Primary outcomes were discharge to community vs a formal postacute care setting (ie, skilled nursing facility, home health care, or inpatient rehabilitation facility) and readmission and mortality within 30, 90, and 180 days of hospital discharge.

**Results:**

Among 2 044 231 hospitalizations from 2011 to 2015, 1 538 888 (75.2%; mean [SD] age, 80.4 [8.3] years; 866 540 [56.3%] women) were among patients from urban counties, 322 360 (15.8%; mean [SD] age, 79.6 [8.1] years; 175 806 [54.5%] women) were among patients from urban-adjacent rural counties, and 182 983 (9.0%; mean [SD] age, 79.8 [8.1] years; 98 775 [54.0%] women) were among patients from urban-nonadjacent rural counties. The probability of discharge to community without postacute care did not differ by rurality. However, compared with patients from urban counties, patients from the most rural counties were more frequently discharged to a skilled nursing facility (adjusted difference, 3.5 [95% CI, 2.8-4.3] percentage points), while discharge to an inpatient rehabilitation facility was less common among patients from rural counties than among those from urban counties (urban vs urban-adjacent rural: adjusted difference, –1.9 [95% CI, –2.4 to –1.4] percentage points; urban vs urban-nonadjacent rural: adjusted difference, –1.8 [95% CI, –2.4 to –1.2] percentage points) as was discharge to home health care (urban vs urban-adjacent rural: adjusted difference, –1.7 [95% CI, –2.3 to –1.2] percentage points; urban vs urban-nonadjacent rural: adjusted difference, –2.4 [95% CI, –3.0 to –1.8]). For patients from the most rural counties, adjusted 30-day readmission rates were 0.4 (95% CI, 0.2-0.6) percentage points higher than those of patients from urban counties among those who were discharged to the community but 0.3 (95% CI, –0.6 to –0.1) percentage points lower among patients receiving postacute care. Adjusted 30-day mortality rates were 0.4 (95% CI, 0.3-0.5) percentage points higher for patients from the most rural counties discharged to the community and 2.0 (95% CI, –1.7 to 2.3) percentage points higher among those receiving postacute care. Rural-urban differences in 90-day and 180-day outcomes were similar.

**Conclusions and Relevance:**

These findings suggest that rates of discharge to the community and postacute care settings were similar among patients from rural and urban counties. Rural-urban differences in mortality following discharge were much larger for patients receiving postacute care compared with patients discharged to the community setting. Improving postacute care in rural areas may reduce rural-urban disparities in patient outcomes.

## Key Points

**Question:**

Do postacute care utilization and outcomes differ between patients from rural vs urban counties who have been hospitalized for stroke, hip fracture, chronic obstructive pulmonary disease, congestive heart failure, or pneumonia?

**Findings:**

In this cohort study of 2 044 231 US patients 66 years and older, overall postacute care utilization was similar among patients from rural and urban counties. Adjusted 30-day postdischarge mortality rates were higher among patients from rural counties than among those from urban counties, and adjusted rural-urban differences were larger among patients discharged to formal postacute care settings than among those discharged to the community setting.

**Meaning:**

These findings suggest that improving postacute care in rural areas may reduce rural-urban disparities in patient outcomes after hospitalization.

## Teaser

This cohort study examines differences among patients from urban vs rural counties in outcomes after discharge from acute care by discharge destination.

## Introduction

Approximately 60 million people in the United States reside in rural areas, accounting for 19% of the US population overall and approximately one-quarter of all Medicare beneficiaries.^1,2^ Several studies have found that compared with urban and suburban areas, rural communities have less access to health care services, such as primary and secondary ambulatory care and hospital care.^3,4,5^ Less is known about the use of postacute care in rural America. Access to high-quality postacute care may be particularly important for patients in rural areas for several reasons. First, rural communities are composed of residents who are disproportionately older and affected by chronic illnesses compared with residents of nonrural communities.^3^ Second, the quality of postacute care and type of posthospital setting are associated with clinical and health service utilization outcomes.^6,7^ Third, the primary conditions associated with the increasing rural-urban disparity in life expectancy commonly require hospitalization and subsequent postacute care.^8,9^

There are several reasons postacute care may be less available or lower in quality in rural areas. Owing to low population density, the financial viability of some forms of postacute care may be reduced in rural markets. For example, the distances home health care personnel must travel to residences may be longer and thus more resource intensive. Institutional postacute care facilities, such as inpatient rehabilitation facilities (IRFs) and skilled nursing facilities (SNFs), have high fixed costs and may be too costly to operate without a critical mass of patients. The higher costs of postacute care in combination with lower population density in rural areas may reduce competition between fewer health care institutions, which in turn may lower access to postacute care, exacerbate poor quality, and adversely affect patient outcomes. Despite these concerns, there is limited knowledge of discrepancies in the use and outcomes of postacute care between individuals residing in rural vs urban areas. The absence of such information may not only complicate efficient and informed hospital discharge planning but also attenuate policy efforts to ameliorate the increasing rural-urban disparity in health outcomes

The objective of this study was to examine differences in the frequency, type, and duration of postacute care utilization among rural and urban Medicare beneficiaries. We also investigate the role of postacute care in rural-urban disparities in patient outcomes by comparing rural-urban differences in readmission and mortality rates among individuals discharged to postacute care and among individuals discharged to the community.

## Methods

Brown University’s institutional review board approved the study protocol and waived the requirement for participant informed consent owing to the infeasibility of acquiring consent in claims data. Data analyses were conducted between October 1, 2018, and May 30, 2019. This study followed the Strengthening the Reporting of Observational Studies in Epidemiology (STROBE) reporting guideline.

### Data Sources

The primary sources of individual-level data were the Medicare Beneficiary Summary file and the Medicare Provider Analysis and Review database. The Medicare Beneficiary Summary file contains data on demographic characteristics, managed care (ie, Medicare Advantage) participation, and dates of death for all enrolled Medicare beneficiaries. The Medicare Provider Analysis and Review database includes all fee-for-service (FFS) claims related to inpatient, SNF, home health, and hospice services, containing admitting diagnosis and dates of services. We supplemented claims and enrollment data with SNF, IRF, and home health patient assessment data to ascertain postacute care use with more certainty.^10,11,12^ County-level data were obtained from the Area Health Resource File.

### Study Sample

Our sample consisted of Medicare FFS beneficiaries who were hospitalized between January 1, 2011, and September 30, 2015, with a primary diagnosis of stroke, hip or femur fracture without joint replacement, chronic obstructive pulmonary disease, congestive heart failure, or pneumonia. These conditions have been associated with increasing rural-urban differences in mortality rates and are commonly treated in postacute care.^8,9,13,14^ Diagnoses were identified using the primary *International Classification of Diseases, Ninth Revision* (*ICD-9*)^15^ code and the Medicare Severity Diagnosis Related Group (MS-DRG) of the hospital claim in combination (eTable 1 in the Supplement). A 1-year lookback period was needed to evaluate the complete set of exclusion criteria, which were being younger than 66 years; receiving care from an acute care hospital, SNF, IRF, or home health service in the year preceding the index hospitalization; being enrolled in Medicare Advantage at the time of hospitalization or during the preceding year; having a claim discharge code of left against medical advice or planned acute care readmission; being discharged after September 30, 2015, for which follow-up is censored; and living outside of the 48 contiguous United States.

### Rural and Urban Classifications

Individuals were classified as rural- or urban-dwelling based on the 2013 Rural-Urban Continuum Code^16^ assigned to their county of residence. Rural-Urban Continuum Codes were developed by the US Department of Agriculture to distinguish counties according to population size and proximity to nearby metropolitan areas. Codes range from 1 to 9, with higher values representing decreasing levels of urbanization. Based on prior work,^17,18^ we classified residents of counties with codes of 1 to 3 as urban and residents of counties with codes of 4 to 9 as rural. Because proximity to a metropolitan area may be associated with access to health services, rural residents were divided further into those residing in counties adjacent to metropolitan areas (codes 4, 6, and 8) and those residing in counties not adjacent to metropolitan areas (codes 5, 7, and 9).

### Outcome Measures

The primary outcomes of this study were posthospital discharge setting, hospital readmission, and postdischarge mortality. We combined claims and postacute care assessment data with dates of death to classify individuals as being in 1 of 6 settings on each day of the 90-day postdischarge period. Settings were classified as community without home health; community with home health; SNF, nursing home, or swing bed; IRF; acute or other inpatient care; and dead. Unplanned hospital readmission, defined as any general acute care or critical access hospital admission, and mortality were assessed at 30 days after discharge.^19^

We included as additional outcomes readmission and mortality at 90 days because of the increasing attention this period of care receives from federal policy initiatives and because this period encompasses most postacute care episodes. We used mortality within 180 days of discharge as a long-term outcome measure.

### Statistical Analysis

We used multinomial logistic regression to examine whether the probability of initial discharge to a given postacute care or non–postacute care setting differed between patients from rural vs urban counties. To compare the duration and course of postacute care utilization, we also estimated the adjusted probability of being in any given setting on each day after hospital discharge until 90 days (via 90 separate models, 1 for each day). We provided separate trajectories for individuals discharged to an institutional postacute care setting and for individuals discharged to the community with home health, which allowed us to examine rural-urban differences in home health use after institutional postacute care. We then used logistic regression to assess whether rural residence was associated with readmission or mortality, conditional on postacute care utilization. Specifically, we fit separate models for individuals discharged to the community and for individuals discharged to postacute care, excluding those who were discharged to a different hospital setting or to hospice services or who were discharged dead.

All adjusted models included individual-level covariates: age, sex, race/ethnicity, Medicaid eligibility, Elixhauser Comorbidity Index score,^20^ hospital length of stay, admitting diagnosis, intensive care unit admission, and major US Census region (ie, Northeast, Midwest, South, or West). Point estimates are presented as adjusted mean probabilities and percentage point differences between patients from rural vs urban counties, which were calculated using the marginal standardization form of predictive margins. Data were analyzed with Stata/MP statistical software version 15.1 (StataCorp). Null hypotheses were tested assuming a 2-sided type I error probability of .05.

We conducted 3 robustness checks on our clinical outcomes analyses. First, we excluded individuals who were admitted to critical access hospitals. Second, we included several county-level socioeconomic controls to our adjusted models: the proportions of residents who were white, college-educated, unemployed, without health insurance, or impoverished. Third, we stratified analyses by primary diagnosis.

## Results

We identified 2 044 231 index admissions for FFS Medicare beneficiaries who met our study inclusion criteria, including 1 538 888 (75.2%; mean [SD] age, 80.4 [8.3] years; 866 540 [56.3%] women) admissions for residents of urban counties, 322 360 (15.8%; mean [SD] age, 79.6 [8.1] years; 175 806 [54.5%] women) admissions for residents of rural counties adjacent to an urban county, and 182 983 (9.0%; mean [SD] age, 79.8 [8.1] years; 98 775 [54.0%] women) admissions by residents of rural counties not adjacent to an urban county. Compared with patients from urban counties, patients from rural counties were less likely to be nonwhite, including 23 755 patients (7.4%) from urban-adjacent rural counties and 11 314 patients (6.2%) from urban-nonadjacent rural counties, compared with 192 378 patients (12.5%) from urban counties, and more likely to be eligible for Medicaid, including 56 756 patients (17.6%) from urban-adjacent rural counties and 33 266 patients (18.2%) from urban-nonadjacent rural counties, compared with 211 189 patients (13.7%) from urban counties (Table 1). Patients from rural counties were more frequently hospitalized for pneumonia (urban-adjacent rural counties: 94 832 patients [29.4%]; urban-nonadjacent rural counties: 58 883 patients [32.2%]; urban counties: 391 908 patients [25.4%]) or hip or femur fracture (urban-adjacent rural counties: 61 295 patients [19.0%]; urban-nonadjacent rural counties: 34 697 patients [19.0%]; urban counties: 252 098 patients [16.4%]); were less frequently hospitalized for stroke (urban-adjacent rural counties: 68 410 patients [21.2%]; urban-nonadjacent rural counties: 36 754 patients [20.1%]; urban counties: 381 934 patients [24.8%]); and were less likely to have been admitted to an intensive care unit (urban-adjacent rural counties: 70 997 patients [22.0%]; urban-nonadjacent rural counties: 36 617 patients [20.0%]; urban counties: 442 027 patients [28.7%]). Patients from rural counties were less likely to be located in the Northeast (urban-adjacent rural counties: 37 186 patients [11.7%]; urban-nonadjacent rural counties: 11 744 patients [6.4%] urban counties: 338 908 patients [22.0%]) and more likely to be located in the Midwest (urban-adjacent rural counties: 108 698 [33.7%]; urban-nonadjacent rural counties: 71 584 patients [39.1%] urban counties: 349 898 patients [22.7%]). Patients from urban-adjacent rural counties in particular were disproportionally located in the South compared with patients in urban-nonadjacent rural counties (145 901 patients [45.3%] vs 70 207 patients [38.4%]) or patients from urban counties (589 026 patients [38.3%]). However, patients from urban-adjacent rural counties were less present in the West compared with patients from urban-nonadjacent counties (30 575 patients [9.5%] vs 29 448 patients [16.1%]) or patients from urban counties (261 056 patients [17.0%]). Patient characteristics stratified by rurality and postdischarge setting are listed in eTable 2 in the Supplement.

**Table 1.** Study Cohort Characteristics by Residential Location

Characteristic	Patients, No. (%)		
	Urban (n = 1 538 888)	Rural	
		Urban Adjacent (n = 322 360)	Urban Nonadjacent (n = 182 983)
Age, mean (SD), y	80.4 (8.3)	79.6 (8.1)	79.8 (8.1)
Women	866 540 (56.3)	175 806 (54.5)	98 775 (54.0)
Nonwhite race	192 378 (12.5)	23 755 (7.4)	11 314 (6.2)
Medicaid eligible	211 189 (13.7)	56 576 (17.6)	33 266 (18.2)
Census region			
Northeast	338 908 (22.0)	37 186 (11.7)	11 744 (6.4)
Midwest	349 898 (22.7)	108 698 (33.7)	71 584 (39.1)
South	589 026 (38.3)	145 901 (45.3)	70 207 (38.4)
West	261 056 (17.0)	30 575 (9.5)	29 448 (16.1)
Primary diagnosis			
Pneumonia	391 255 (25.4)	94 832 (29.4)	58 883 (32.2)
Stroke	381 934 (24.8)	68 410 (21.2)	36 754 (20.1)
Heart failure	324 745 (21.1)	63 653 (19.7)	33 704 (18.4)
Hip or femur fracture repair	252 098 (16.4)	61 295 (19.0)	34 697 (19.0)
COPD	188 856 (12.3)	34 170 (10.6)	18 945 (10.4)
Length of stay, mean (SD), d	5.4 (3.5)	5.2 (3.3)	5.2 (3.2)
ICU admission	442 027 (28.7)	70 997 (22.0)	36 617 (20.0)
Elixhauser Comorbidity Index score, mean (SD)	3.0 (1.7)	2.8 (1.7)	2.8 (1.6)

Abbreviations: COPD, chronic obstructive pulmonary disease; ICU, intensive care unit.

Patients did not differ by rurality in their likelihood of being discharged to the community without home health (Table 2). However, patients from rural counties were more likely to be discharged to an SNF, while patients from urban counties were more likely to be discharged to an IRF or to the community with home health. Among patients from urban counties, 197 151 (12.8%) were discharged to the care of a home health agency, 319 532 (20.8%) were discharged to an SNF or nursing home, and 147 257 (9.6%) were discharged to an IRF. Among patients from urban-adjacent rural counties, 33 982 (10.5%) were discharged to the care of a home health agency (adjusted difference, –1.7 [95% CI, –2.3 to –1.2] percentage points), 69 270 (21.5%) were discharged to a SNF (adjusted difference, 3.0 [95% CI, 2.4-3.6] percentage points), and 21 073 (6.5%) were discharged to an IRF (adjusted difference, –1.9 [95% CI, –2.4 to –1.4] percentage points). Among patients from urban-nonadjacent rural counties, 17 590 (9.6%) were discharged to the care of a home health agency (adjusted difference, –2.4 [95% CI, –3.0 to –1.8] percentage points), 40 649 (22.2%) were discharged to an SNF (adjusted difference, 3.5 [95% CI, 2.8-4.3] percentage points), and 11 330 (6.2%) were discharged to an IRF (adjusted difference, –1.8 [95% CI, –2.4 to –1.2] percentage points).

**Table 2.** Fraction of Patients Discharged to Postacute Care and Other Settings by Residential Location

Discharge Outcome	Patients, No. (%)			Difference (95% CI)			
	Urban	Rural		Urban Adjacent Rural vs Urban		Urban Nonadjacent Rural vs Urban	
		Urban Adjacent	Urban Nonadjacent	Unadjusted	Adjusted^a^	Unadjusted	Adjusted^a^
Community	780 648 (50.7)	179 214 (55.6)	103 036 (56.3)	4.9 (4.0 to 5.7)	–0.2 (–0.7 to 0.4)	5.6 (4.6 to 6.5)	–0.1 (–0.8 to 0.6)
Home health	197 151 (12.8)	33 982 (10.5)	17 590 (9.6)	–2.3 (–2.9 to –1.7)	–1.7 (–2.3 to –1.2)	–3.2 (–3.8 to –2.6)	–2.4 (–3.0 to –1.8)
SNF	319 532 (20.8)	69 270 (21.5)	40 649 (22.2)	0.7 (–0.1 to 1.5)	3.0 (2.4 to 3.6)	1.5 (0.6 to 2.3)	3.5 (2.8 to 4.3)
IRF	147 257 (9.6)	21 073 (6.5)	11 330 (6.2)	–3.0 (–3.6 to –2.5)	–1.9 (–2.4 to –1.4)	–3.4 (–3.9 to –2.8)	–1.8 (–2.4 to –1.2)
Other^b^	49 163 (3.2)	8642 (2.7)	4298 (2.3)	–0.5 (–0.7 to –0.4)	–0.2 (–0.3 to –0.1)	–0.9 (–1.0 to –0.7)	–0.5 (–0.6 to –0.3)
Dead	45 137 (2.9)	10 179 (3.2)	6080 (3.3)	0.2 (0.1 to 0.3)	0.9 (0.8 to 1.1)	0.4 (0.3 to 0.5)	1.3 (1.2 to 1.5)

Abbreviations: IRF, inpatient rehabilitation facility; SNF, skilled nursing facility.

^a^ Adjusted results were derived from a multinomial logistic regression model adjusted for age, female sex, nonwhite race, Medicaid eligibility, Elixhauser Comorbidity Index score, hospital length of stay, admitting diagnosis, intensive care unit admission, and census region (ie, Northeast, Midwest, South, or West).

^b^ Includes discharge to another inpatient setting (eg, psychiatric hospital) or to the community on hospice.

Table 3 shows rural-urban differences in rates of adverse outcomes for patients discharged to a postacute care setting and for patients discharged to the community without postacute care. Pooled results are listed in eTable 3 in the Supplement. Among patients discharged to the community, the unadjusted 30-day readmission rate was 9.3% for urban residents, 9.6% for residents of urban-adjacent rural counties, and 9.4% for residents of urban-nonadjacent rural counties. In adjusted analyses, the 30-day readmission rate was 9.2% for residents of urban counties, 9.7% for residents of urban-adjacent rural counties (adjusted difference, 0.5 [95% CI, 0.3-0.6] percentage points), and 9.7% for residents of urban-nonadjacent rural counties (adjusted difference, 0.4 [95% CI, 0.2-0.6] percentage points). Among patients discharged to postacute care, the unadjusted 30-day readmission rate was 12.0% for patients in urban counties, 11.7% for residents of urban-adjacent rural counties, and 11.1% for residents of urban-nonadjacent rural counties. In adjusted analyses, the 30-day readmission rate was 11.9% for residents of urban counties, 11.9% for residents of urban-adjacent rural counties (adjusted difference, –0.1 [95% CI, –0.3 to 0.2] percentage points), and 11.6% for residents of urban-nonadjacent rural counties (adjusted difference, –0.3 [95% CI, –0.6 to –0.1] percentage points).

**Table 3.** Rural-Urban Differences in Rates of Adverse Outcomes Stratified by Postdischarge Setting

Outcome	Community			Postacute Care^a^		
	Patients, No.	Rate, %		Patients, No.	Rate, %	
		Unadjusted	Adjusted^b^		Unadjusted	Adjusted^b^
30-d readmission						
Urban	71 399	9.3	9.2	75 916	12.0	11.9
Rural						
Urban adjacent	16 883	9.6	9.7	13 648	11.7	11.9
Urban nonadjacent	9547	9.4	9.7	7218	11.1	11.6
Difference						
Urban-adjacent rural vs urban, percentage points (95% CI)		0.3 (0.1 to 0.5)	0.5 (0.3 to 0.6)		–0.3 (–0.6 to –0.1)	–0.1 (–0.3 to 0.2)
Urban-nonadjacent rural vs urban, percentage points (95% CI)		0.2 (–0.1 to 0.4)	0.4 (0.2 to 0.6)		–0.9 (–1.3 to –0.6)	–0.3 (–0.6 to –0.1)
30-d mortality						
Urban	11 081	1.4	1.4	31 149	4.7	4.6
Rural						
Urban adjacent	2949	1.7	1.7	7228	5.8	6.1
Urban nonadjacent	1812	1.8	1.8	4480	6.4	6.6
Difference						
Urban-adjacent rural vs urban (95% CI)		0.2 (0.2 to 0.3)	0.4 (0.3 to 0.4)		1.1 (0.9 to 1.3)	1.5 (1.3 to 1.7)
Urban-nonadjacent rural vs urban (95% CI)		0.4 (0.2 to 0.4)	0.4 (0.3 to 0.5)		1.8 (1.5 to 2.0)	2.0 (1.7 to 2.3)
90-d readmission						
Urban	129 400	17.3	17.2	123 334	20.8	20.7
Rural						
Urban adjacent	30 320	17.7	17.8	22 218	20.3	20.7
Urban nonadjacent	17 030	17.3	17.8	11 935	19.7	20.4
Difference						
Urban-adjacent rural vs urban (95% CI)		0.4 (0.2 to 0.7)	0.6 (0.4 to 0.9)		–0.5 (–0.9 to –0.1)	–0.1 (–0.4 to 0.3)
Urban-nonadjacent rural vs urban (95% CI)		0.1 (–0.3 to 0.4)	0.5 (0.2 to 0.9)		–1.2 (–1.6 to –0.7)	–0.3 (–0.8 to 0.1)
90-d mortality						
Urban	32 334	4.1	4.1	71 480	10.8	10.7
Rural						
Urban adjacent	8253	4.6	4.8	15 092	12.1	12.7
Urban nonadjacent	4839	4.7	4.8	8870	12.8	13.1
Difference						
Urban-adjacent rural vs urban (95% CI)		0.5 (0.3 to 0.6)	0.7 (0.6 to 0.8)		1.4 (1.1 to 1.7)	2.0 (1.7 to 2.3)
Urban-nonadjacent rural vs urban (95% CI)		0.6 (0.4 to 0.7)	0.8 (0.6 to 0.9)		2.0 (1.6 to 2.4)	2.4 (2.0 to 2.9)
180-d mortality						
Urban	57 754	7.4	7.3	107 384	16.2	16.0
Rural						
Urban adjacent	14 388	8.0	8.3	21 767	17.5	18.1
Urban nonadjacent	8359	8.1	8.3	12 635	18.2	18.6
Difference						
Urban-adjacent rural vs urban (95% CI)		0.6 (0.4 to 0.8)	1.0 (0.8 to 1.2)		1.3 (1.0 to 1.7)	2.1 (1.8 to 2.5)
Urban-nonadjacent rural vs urban (95% CI)		0.7 (0.5 to 1.0)	1.0 (0.8 to 1.3)		2.0 (1.6 to 2.4)	2.6 (2.1 to 3.0)

^a^ Includes patients discharged to a skilled nursing facility, inpatient rehabilitation facility, or to the community with home health.

^b^ Adjusted results were derived from logistic regression models adjusted for age, female sex, nonwhite race, Medicaid eligibility, Elixhauser comorbidity index score, hospital length of stay, admitting diagnosis, intensive care unit admission, and census region (ie, Northeast, Midwest, South, or West).

Rural-urban differences in 30-day mortality rates are also described in Table 3. Among patients discharged to the community, the unadjusted 30-day mortality rate was 1.4% for residents of urban counties, 1.7% for residents of urban-adjacent rural counties, and 1.8% for residents of urban-nonadjacent rural counties. In adjusted analyses, the 30-day mortality rate was 1.4% for residents or urban counties, 1.7% for residents of urban-adjacent rural counties (adjusted difference, 0.4 [95% CI, 0.3-0.4] percentage points), and 1.8% for residents of urban-nonadjacent rural counties (adjusted difference, 0.4 [95% CI, 0.3-0.5] percentage points). Among patients discharged to postacute care, the unadjusted 30-day mortality rate was 4.7% for residents or urban counties, 5.8% for residents of urban-adjacent rural counties, and 6.4% for residents of urban-nonadjacent rural counties. In adjusted analyses, the 30-day mortality rate was 4.6% for residents of urban counties, 6.1% for residents of urban-adjacent rural counties (adjusted difference, 1.5 [95% CI, 1.3-1.7] percentage points), and 6.6% for residents of urban-nonadjacent rural counties (adjusted difference, 2.0 [95% CI, 1.7-2.3] percentage points). Rural-urban differentials in rates of 90-day and 180-day outcomes were similar to 30-day outcomes (Table 3). Sensitivity and diagnosis-specific analyses (eTable 4 and eFigures 1-5 in the Supplement) also generated consistent findings.

**Figure 1.**
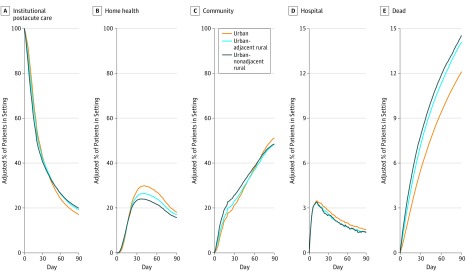
90-Day Heath Care Utilization and Mortality Trajectories After Discharge to Institutional Postacute Care by Rurality The adjusted proportion of individuals in each setting on each day was derived from multinomial logistic regression models adjusted for age, female sex, nonwhite race, Medicaid eligibility, Elixhauser Comorbidity Index score, hospital length of stay, admitting diagnosis, intensive care unit admission, and census region (ie, Northeast, Midwest, South, or West). Institutional postacute care includes patients in a skilled nursing facility, nursing home, swing bed, or inpatient rehabilitation facility. Hospital includes patients admitted to a general acute care hospital, critical access hospital, or other inpatient setting (eg, psychiatric hospital).

Figure 1 illustrates the adjusted proportion of patients who were using postacute or other health care in the community without home health and who died during the 90-day period after initial discharge to an institutional postacute care setting. The corresponding estimated rural-urban differences in postdischarge trajectories are illustrated in eFigure 6 in the Supplement. The proportion of patients in institutional postacute care declined during the postdischarge period; however, patients from rural counties were more likely to remain institutionalized at 90 days after discharge (Figure 1A). In contrast, patients from rural counties were less likely to use follow-up home health care (Figure 1B). Accordingly, patients from rural counties were more likely to reside in the community without home health approximately 30 days after discharge (Figure 1C), but by 90 days after discharge, they were less likely to reside in the community without home health, as they were more likely than patients from urban counties to die by this point (Figure 1E). The daily proportion of patients in an acute care or other inpatient setting was consistently low (Figure 1D), and was marginally lower among patients from rural counties.

**Figure 2.**
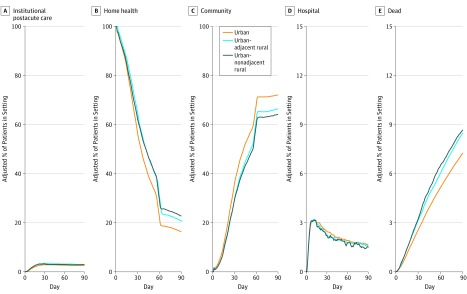
90-Day Heath Care Utilization and Mortality Trajectories After Discharge to Home With Home Health by Rurality The adjusted proportion of individuals in each setting on each day was derived from multinomial logistic regression models adjusted for age, female sex, nonwhite race, Medicaid eligibility, Elixhauser Comorbidity Index score, hospital length of stay, admitting diagnosis, intensive care unit admission, and census region (ie, Northeast, Midwest, South, or West). Institutional postacute care includes patients in a skilled nursing facility, nursing home, swing bed, or inpatient rehabilitation facility. Hospital includes patients admitted to a general acute care hospital, critical access hospital, or other inpatient setting (eg, psychiatric hospital).

Figure 2 shows the adjusted proportion of patients in each postdischarge setting throughout the 90-day follow-up period, specifically among individuals initially discharged to the care of a home health agency. The corresponding estimated rural-urban differences in trajectories are presented in eFigure 7 in the Supplement. The proportion of patients using institutional postacute care, acute care, or other inpatient care was consistently low throughout the postdischarge period (Figure 2A and D). The proportion of patients using home health care decreased with follow-up time, particularly at day 60, when home health benefits typically need to be renewed (Figure 2B). In comparison with patients in urban counties, patients in rural counties had longer home health episodes and died more frequently (Figure 2E).

## Discussion

In this cohort study of FFS Medicare beneficiaries, we examined differences in postacute care utilization and outcomes between residents of rural vs urban areas who were hospitalized for stroke, hip or femur fracture, chronic obstructive pulmonary disease, heart failure, or pneumonia. We found that patients from rural and urban counties were equally likely to be discharged to a formal postacute care setting; however, the type and course of postacute care differed according to rurality. Patients from rural counties were more likely to be discharged to an SNF, while patients from urban counties were more likely to be discharged to an IRF or to the community with home health care. After discharge to an institutional postacute care setting, patients from rural counties were less likely to receive follow-up home health care and were more likely to remain institutionalized. Additionally, patients from rural counties discharged to the community with home health had longer home health episodes compared with patients from urban counties.

We also found that rural residence was associated with increased risk of mortality and that this risk was substantially larger for patients discharged to a postacute care setting. For example, the adjusted percentage point difference in 30-day mortality rates between patients from urban-nonadjacent rural counties vs those from urban counties was 0.4 for patients discharged to community and 2.0 among patients discharged to postacute care. These results were robust in a number of sensitivity analyses as well as diagnosis-specific analyses. Rural residence was associated with a modestly increased risk of 30-day readmission among patients discharged to the community. Among patients discharged to postacute care, 30-day readmission rates did not differ between patients from urban counties vs patients from urban-adjacent rural counties. However, readmission rates were marginally lower for patients discharged to postacute care from the urban-nonadjacent rural counties. Reduced access to acute care in rural areas for patients who are more seriously ill may be one explanation for readmission rates that were similar or lower to urban rates as well as higher mortality rates. This is an important area to investigate further, particularly because there has recently been an increase in rural hospital closures.^5^

To our knowledge, few prior studies have examined differences in postacute care between patients from urban vs rural areas. Like these other studies, we also found that patients from rural counites were less likely to be discharged to the care of a home health agency.^21^ However, our study diverges from previous work in important ways. First, our analysis was conducted on a cohort of patients with high-risk conditions that are often treated in postacute care. Other studies have examined rural-urban differences in postacute care among patient populations that are either narrowly defined (eg, joint replacement) or very heterogeneous, including data from patients who may not require postacute care.^21,22,23^ Second, we estimated and compared rural-urban differences in adverse outcomes between patients discharged to a formal postacute care setting and patients discharged to the community. Additionally, while other studies have examined rural-urban differences in initial hospital discharge setting, our study examines the continuum of postacute care services as well as clinical outcomes longitudinally. This analysis found important differences in the intensity of institutional postacute care and home health services used by patients from rural counties, as well as large differences in short-term and later mortality after discharge to postacute care.

Although there may be several explanations for increased mortality associated with postacute care among patients from rural areas, there are a few that may be central. First, differences between patients from rural vs urban counties in unobserved comorbidity may be larger in the postacute care setting. Second, postacute care delivered to patients in rural areas may be lower quality or differ in other important ways compared with postacute care delivered to patients in urban areas. Third, patients in rural areas may receive lower quality hospital care when discharged to postacute care but not when discharged to the community. Additionally, a combination of these factors may be involved; for instance, rural hospitals may have difficulty treating patients who are severely ill. Owing to this study’s observational design, we are unable to distinguish among these potential mechanisms. Nevertheless, enhancing clinical care for rural communities does not depend on whether its need is rooted in the quality of the health care provided or in increased patient need.

Many of the Centers for Medicare & Medicaid Services’ current and planned policy initiatives target postacute care as an area to reduce costs and improve value-based care. However, many of these initiatives do not involve rural hospitals and postacute care institutions. Given the higher mortality rates after hospitalization among patients in rural areas, our findings suggest that improving the effectiveness of postacute care for patients in rural areas should be an important focus for policy makers, clinicians, and other stakeholders.

### Limitations

This study has limitations. Our cohort consisted of individuals without prior acute or postacute care, with 1 of 5 primary diagnoses that had matching *ICD-9* and MS-DRG codes. This enabled us to estimate rural-urban differences in postacute care among patients with similar clinical presentations at the time of hospitalization and who were previously stable. However, our results may not be generalizable to patients with worse health status who would be unlikely to meet these inclusion criteria or Medicare beneficiaries enrolled in Medicare Advantage. Additionally, despite the cohort’s homogeneity, it is possible that there are unobserved differences between patients from rural vs urban counties that may affect our results. Potential unobserved rural-urban differences in patient- or hospital-level factors would need to be asymmetric between patients discharged to the community and patients discharged to postacute care to account for the greater mortality rates among patients from rural areas in postacute care. Additionally, we did not examine care process or other measures of hospital and postacute care quality.

## Conclusions

Our findings suggest that the overall use of formal postacute care did not differ among rural and urban Medicare FFS beneficiaries. However, we found differences in the course and duration of postacute care. Patients from rural counties were less likely to use home health care, even after discharge from an institutional postacute care setting, and used postacute care for longer periods. Rural-urban differences in postdischarge mortality were larger among patients who received postacute care compared with patients who were discharged to the community. Increased attention to postacute care in rural areas may improve patients’ outcomes.

## Notes

Supplement. **eTable 1.** Inclusionary Diagnoses and Medicare Severity Diagnosis Related Groups **eTable 2.** Study Cohort Characteristics by Residential Location and Postdischarge Setting **eTable 3.** Overall Rural-Urban Differences in Adjusted Rates of Adverse Outcomes **eTable 4.** Sensitivity Estimates of Adjusted Rural-Urban Differences in Rates of Adverse Outcomes Stratified by Postdischarge Setting **eFigure 1.** Adjusted Rural-Urban Differences in Rates of Adverse Outcomes Among Individuals Admitted for Stroke **eFigure 2.** Adjusted Rural-Urban Differences in Rates of Adverse Outcomes Among Individuals Admitted for Chronic Obstructive Pulmonary Disease **eFigure 3.** Adjusted Rural-Urban Differences in Rates of Adverse Outcomes Among Individuals Admitted for Heart Failure **eFigure 4.** Adjusted Rural-Urban Differences in Rates of Adverse Outcomes Among Individuals Admitted for Hip Fracture **eFigure 5.** Adjusted Rural-Urban Differences in Rates of Adverse Outcomes Among Individuals Admitted for Pneumonia **eFigure 6.** Rural-Urban Differences in 90-Day Health Care Utilization and Mortality Trajectories After Discharge to Institutional Postacute Care **eFigure 7.** Rural-Urban Differences in 90-Day Health Care Utilization and Mortality Trajectories After Discharge to Home With Home Health
