# Supplementary material for: Assessment of Rural-Urban Differences in Postacute Care Utilization and Outcomes Among Older US Adults
Source: JAMA Netw Open. 2020 Jan 8;3(1):e1918738. doi: 10.1001/jamanetworkopen.2019.18738 (PMC6991315; doi:10.1001/jamanetworkopen.2019.18738)
Supplement: Supplement. — eTable 1. Inclusionary Diagnoses and Medicare Severity Diagnosis Related Groups eTable 2. Study Cohort Characteristics by Residential Location and Postdischarge Setting eTable 3. Overall Rural-Urban Differences in Adjusted Rates of Adverse Outcomes eTable 4. Sensitivity Estimates of Adjusted Rural-Urban Differences in Rates of Adverse Outcomes Stratified by Postdischarge Setting eFigure 1. Adjusted Rural-Urban Differences in Rates of Adverse Outcomes Among Individuals Admitted for Stroke eFigure 2. Adjusted Rural-Urban Differences in Rates of Adverse Outcomes Among Individuals Admitted for Chronic Obstructive Pulmonary Disease eFigure 3. Adjusted Rural-Urban Differences in Rates of Adverse Outcomes Among Individuals Admitted for Heart Failure eFigure 4. Adjusted Rural-Urban Differences in Rates of Adverse Outcomes Among Individuals Admitted for Hip Fracture eFigure 5. Adjusted Rural-Urban Differences in Rates of Adverse Outcomes Among Individuals Admitted for Pneumonia eFigure 6. Rural-Urban Differences in 90-Day Health Care Utilization and Mortality Trajectories After Discharge to Institutional Postacute Care eFigure 7. Rural-Urban Differences in 90-Day Health Care Utilization and Mortality Trajectories After Discharge to Home With Home Health [file jamanetwopen-3-e1918738-s001.pdf]

Supplementary Online Content

Kosar CM, Loomer L, Ferdows NB, Trivedi AN, Panagiotou OA, Rahman M. Assessment of rural-urban differences in postacute care utilization and outcomes among older US adults. *JAMA Netw Open*. 2020;3(1):e1918738. doi:10.1001/jamanetworkopen.2019.18738

eTable 1. Inclusionary Diagnoses and Medicare Severity Diagnosis Related Groups

eTable 2. Study Cohort Characteristics by Residential Location and Postdischarge Setting

eTable 3. Overall Rural-Urban Differences in Adjusted Rates of Adverse Outcomes

eTable 4. Sensitivity Estimates of Adjusted Rural-Urban Differences in Rates of Adverse Outcomes Stratified by Postdischarge Setting

eFigure 1. Adjusted Rural-Urban Differences in Rates of Adverse Outcomes Among Individuals Admitted for Stroke

eFigure 2. Adjusted Rural-Urban Differences in Rates of Adverse Outcomes Among Individuals Admitted for Chronic Obstructive Pulmonary Disease

eFigure 3. Adjusted Rural-Urban Differences in Rates of Adverse Outcomes Among Individuals Admitted for Heart Failure

eFigure 4. Adjusted Rural-Urban Differences in Rates of Adverse Outcomes Among Individuals Admitted for Hip Fracture

eFigure 5. Adjusted Rural-Urban Differences in Rates of Adverse Outcomes Among Individuals Admitted for Pneumonia

eFigure 6. Rural-Urban Differences in 90-Day Health Care Utilization and Mortality Trajectories After Discharge to Institutional Postacute Care

eFigure 7. Rural-Urban Differences in 90-Day Health Care Utilization and Mortality Trajectories After Discharge to Home With Home Health

This supplementary material has been provided by the authors to give readers additional information about their work.

© 2020 Kosar CM et al. *JAMA Network Open.*

eTable 1. Inclusionary Diagnoses and Medicare Severity Diagnosis Related Groups

Diagnosis	ICD-9	MS-DRG
Stroke	430, 431, 433.x1, 434.00, 434.01, 434.10, 434.11, 434.90, 434.91, 435.0, 435.1, 435.3, 435.8, 435.9, 436, 997.02	061–068
Hip Fracture	820.xx, 733.14, 733.15, 733.96, 733.97	480–482, 535, 536
COPD	490, 491, 492, 493.22, 496	190–192
Heart Failure	398.91, 402.01, 402.11, 402.91, 404.01, 404.03, 404.11, 404.13, 404.91, 404.93, 428.0, 428.1, 428.20, 428.21, 428.22, 428.23, 428.30, 428.31, 428.32, 428.33, 428.40, 428.41, 428.42, 428.43, 428.9	291–293
Pneumonia	480.xx–486.xx	193–195

© 2020 Kosar CM et al. *JAMA Network Open.*

eTable 2. Study Cohort Characteristics by Residential Location and Postdischarge Setting

	Urban		Rural-A		Rural-NA	
	Community	PAC[b]	Community	PAC[b]	Community	PAC[b]
Characteristic	n=780,648	n=663,940	n=179,214	n=124,325	n=103,036	n=69,569
Age, mean (sd)	78 (7.7)	82.8 (8)	77.5 (7.5)	82.1 (8)	77.7 (7.5)	82.3 (7.9)
Female sex, no. (%)	390,083 (50)	422,410 (64)	88,165 (49)	77,614 (62)	50,297 (49)	42,940 (62)
Non-white race, no. (%)	105,956 (14)	76,602 (12)	14,245 (8)	8,300 (7)	7,007 (7)	3,671 (5)
Medicaid-eligible, no. (%)	107,395 (14)	93,375 (14)	30,917 (17)	22,838 (18)	18,554 (18)	13,003 (19)
Census region, no. (%)						
Northeast	149,634 (19)	169,797 (26)	18,233 (10)	16,900 (14)	5,992 (6)	5,180 (7)
Midwest	184,676 (24)	145,126 (22)	59,993 (33)	42,537 (34)	38,168 (37)	29,403 (42)
South	313,926 (40)	238,432 (36)	83,641 (47)	53,815 (43)	41,953 (41)	24,402 (35)
West	132,412 (17)	110,585 (17)	17,347 (10)	11,073 (9)	16,923 (16)	10,584 (15)
Primary diagnosis, no. (%)						
Pneumonia	259,420 (33)	114,570 (17)	65,161 (36)	25,312 (20)	40,470 (39)	15,940 (23)
Stroke	133,313 (17)	203,495 (31)	23,860 (13)	36,376 (29)	12,671 (12)	19,543 (28)
Heart Failure	194,506 (25)	111,353 (17)	41,128 (23)	19,053 (15)	21,765 (21)	10,098 (15)
Hip/Femur Fracture Repair	7,773 (1)	174,337 (26)	1,745 (1)	31,164 (25)	1,222 (1)	17,088 (25)
COPD	185,636 (24)	60,185 (9)	47,320 (26)	12,420 (10)	26,908 (26)	6,900 (10)
Length of stay, mean (sd)	4.4 (2.4)	6.4 (3.9)	4.4 (2.2)	6.3 (3.7)	4.4 (2.2)	6.2 (3.6)
ICU admission, no. (%)	196,431 (25)	196,820 (30)	33,090 (18)	29,586 (24)	17,043 (17)	15,319 (22)
Elixhauser index, mean (sd)	2.8 (1.6)	3.1 (1.7)	2.7 (1.6)	3 (1.7)	2.6 (1.6)	2.9 (1.7)

Abbreviations: Rural-A=Rural Adjacent; Rural-NA=Rural non-Adjacent; PAC=Postacute Care; no.= number; sd=standard deviation; COPD=Chronic Obstructive Pulmonary Disease; ICU=Intensive Care Unit.

[a]This table excludes patients from the cohort who were discharged dead or to an alternative setting (see Table 2). [b]Individuals discharged to a skilled nursing facility, inpatient rehabilitation facility, or to the community with home health were considered PAC users

© 2020 Kosar CM et al. *JAMA Network Open.*

eTable 3. Overall Rural-Urban Differences in Adjusted Rates of Adverse Outcomes

	30d Outcome		90d Outcomes		180d
Residence	Readmission	Mortality	Readmission	Mortality	Mortality
Urban	10.4	2.8	18.7	7.0	11.2
Rural-A	10.7	3.7	19.1	8.3	12.7
Rural-NA	10.5	3.9	18.9	8.5	12.9
Δ Rural-A – Urban (95% CI)	0.3 (0.1, 0.4)	0.8 (0.7, 1.0)	0.4 (0.2, 0.6)	1.2 (1.1, 1.4)	1.4 (1.2, 1.7)
Δ Rural-NA – Urban (95% CI)	0.1 (-0.1, 0.3)	1.1 (1.0, 1.3)	0.2 (-0.1, 0.5)	1.5 (1.2, 1.7)	1.7 (1.4, 1.9)

Abbreviations: Rural-A=Rural Adjacent; Rural-NA=Rural non-Adjacent; CI=Confidence Interval

[a]Estimates were derived from logistic regression models. Covariates were: age, female sex, non-white race, Medicaid-eligibility, Elixhauser comorbidity index, hospital length of stay, admitting diagnosis, intensive care unit admission, and census region (Northeast, Midwest, South, and West).

© 2020 Kosar CM et al. *JAMA Network Open.*

eTable 4. Sensitivity Estimates of Adjusted Rural-Urban Differences in Rates of Adverse Outcomes Stratified by Postdischarge Setting

Outcome	(1) Non-CAHS[b]		(2) County-level Covariates[c]	
30d Readmission	Community	PAC[d]	Community	PAC[d]
Urban	9.3	11.9	9.3	11.9
Rural-A	9.7	11.9	9.5	11.8
Rural-NA	9.6	11.4	9.5	11.6
Δ Rural-A–Urban (95% CI)	0.4 (0.2, 0.6)	-0.1 (-0.3, 0.2)	0.2 (-0.1, 0.4)	-0.1 (-0.4, 0.2)
Δ Rural-NA–Urban (95% CI)	0.3 (0.1, 0.5)	-0.5 (-0.9, -0.2)	0.2 (-0.1, 0.4)	-0.3 (-0.7, 0.1)
30d Mortality				
Urban	1.4	4.6	1.4	4.8
Rural Adjacent	1.7	5.8	1.6	5.5
Rural non-Adjacent	1.8	5.9	1.7	6.0
Δ Rural-A – Urban (95% CI)	0.3 (0.2, 0.4)	1.2 (1.0, 1.4)	0.2 (0.1, 0.3)	0.8 (0.6, 1.0)
Δ Rural-NA – Urban (95% CI)	0.4 (0.2, 0.5)	1.3 (1.0, 1.6)	0.3 (0.2, 0.4)	1.3 (1.0, 1.5)
90d Readmission				
Urban	17.3	20.7	17.3	20.7
Rural-A	17.9	20.6	17.5	20.5
Rural-NA	17.7	19.9	17.5	20.4
Δ Rural-A – Urban (95% CI)	0.6 (0.4, 0.9)	-0.2 (-0.5, 0.2)	0.2 (-0.1, 0.4)	-0.2 (-0.6, 0.2)
Δ Rural-NA – Urban (95% CI)	0.5 (0.1, 0.8)	-0.8 (-1.3, -0.3)	0.2 (-0.2, 0.5)	-0.3 (-0.8, 0.2)
90d Mortality				
Urban	4.1	10.6	4.2	10.9
Rural Adjacent	4.7	12.2	4.6	11.7
Rural non-Adjacent	4.8	12.2	4.6	12.2
Δ Rural-A – Urban (95% CI)	0.6 (0.5, 0.8)	1.6 (1.3, 1.9)	.4 (0.3, 0.5)	0.8 (0.6, 1.1)
Δ Rural-NA – Urban (95% CI)	0.7 (0.5, 0.8)	1.7 (1.2, 2.1)	.4 (0.3, 0.6)	1.3 (0.9, 1.7)
180d Mortality				
Urban	7.3	15.9	7.4	16.3
Rural Adjacent	8.3	17.7	7.9	17.0
Rural non-Adjacent	8.3	17.5	8.0	17.6
Δ Rural-A – Urban (95% CI)	0.9 (0.7, 1.1)	1.7 (1.4, 2.1)	0.5 (0.3, 0.7)	0.7 (0.4, 1.0)
Δ Rural-NA – Urban (95% CI)	0.9 (0.7, 1.2)	1.6 (1.1, 2.1)	0.5 (0.3, 0.7)	1.2 (0.8, 1.7)

Abbreviations: PAC= Postacute Care; Rural-A=Rural Adjacent; Rural-NA=Rural non-Adjacent; CI=Confidence Interval; CAH=Critical Access Hospital

[a]Adjusted results were derived from logistic regression models. Covariates were: age, female sex, non-white race, Medicaid-eligibility, Elixhauser comorbidity index, hospital length of stay, admitting diagnosis, intensive care unit admission, and census region (Northeast, Midwest, South, and West). [b]Sensitvity analysis (1) excludes patients admitted to critical access hospitals. [c]Sensitivity analysis (2) includes the following county-level covariates: the proportions of residents who were white, college-educated, unemployed, under age 65 without health insurance, and impoverished. [d]Includes individuals discharged to a skilled nursing facility, inpatient rehabilitation facility, or with home health
.

© 2020 Kosar CM et al. *JAMA Network Open.*

eFigure 1. Adjusted[a] Rural-Urban Differences in Rates of Adverse Outcomes Among Individuals Admitted for Stroke

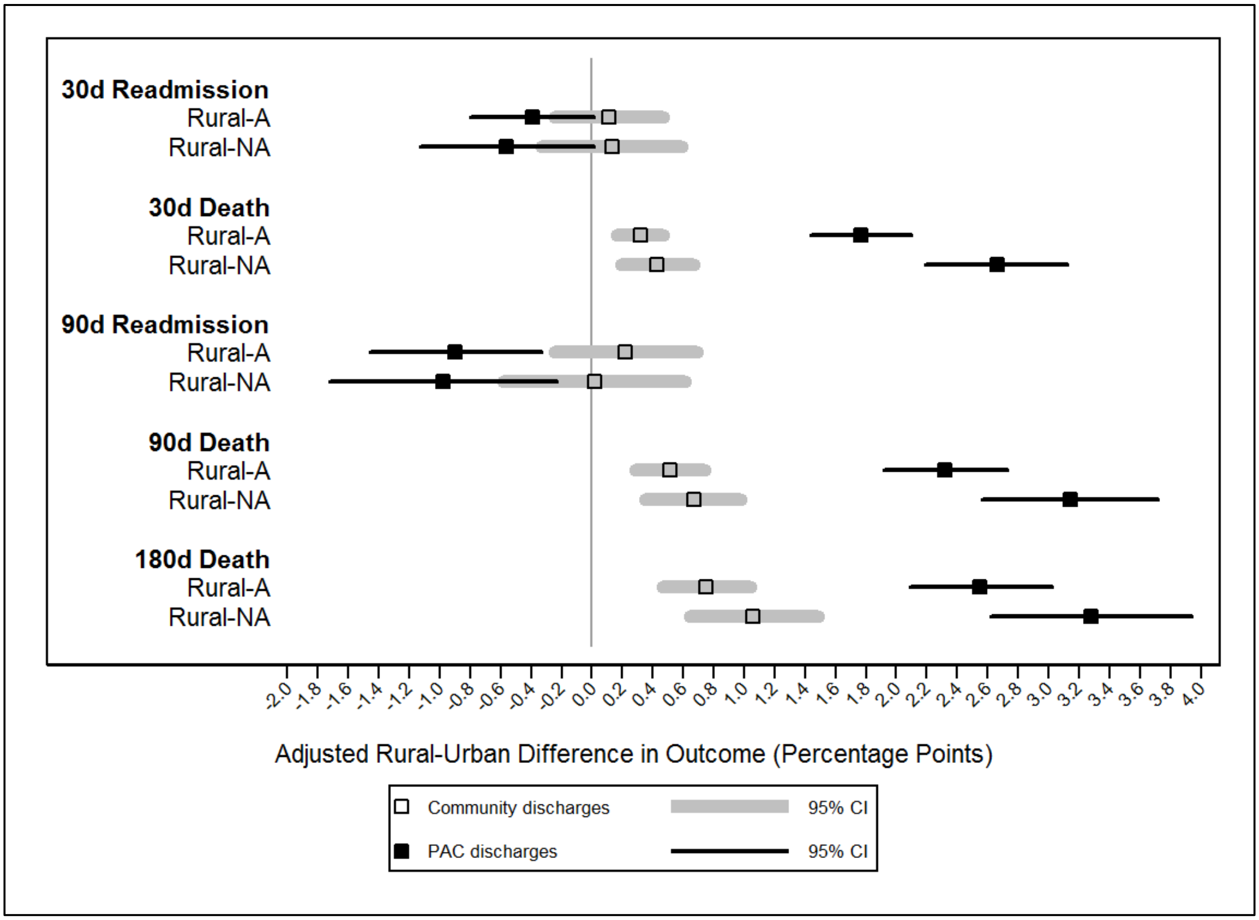

Abbreviations: PAC=Postacute care; Rural-A=Rural Adjacent; Rural-NA=Rural non-Adjacent; CI=Confidence Interval

[a]Estimates were derived from a logistic regression model. Covariates were: age, female sex, non-white race, Medicaid-eligibility, Elixhauser comorbidity index, hospital length of stay, admitting diagnosis, intensive care unit admission, and census region (Northeast, Midwest, South, and West).

© 2020 Kosar CM et al. *JAMA Network Open.*

eFigure 2. Adjusted[a] Rural-Urban Differences in Rates of Adverse Outcomes Among Individuals Admitted for Chronic Obstructive Pulmonary Disease

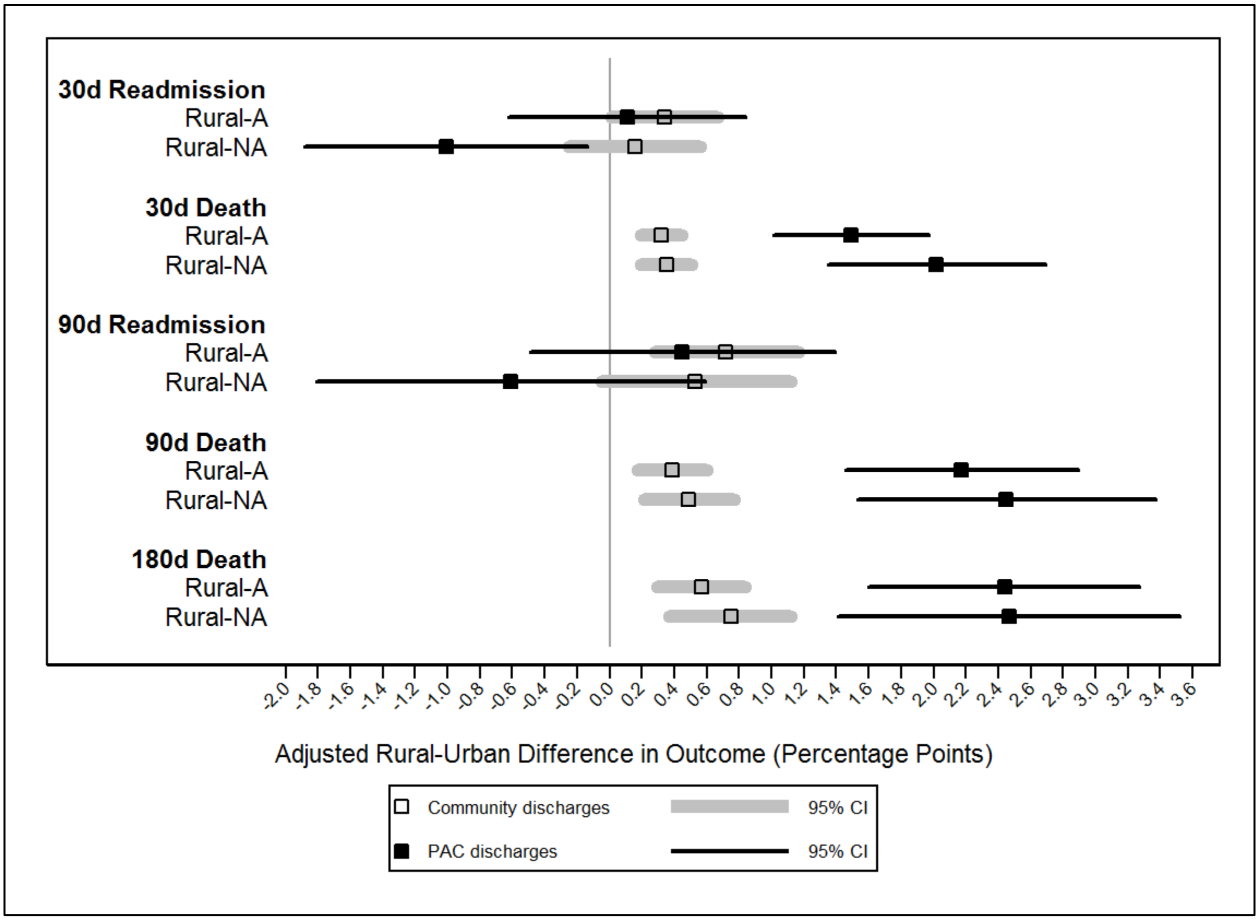

Abbreviations: PAC=Postacute care; Rural-A=Rural Adjacent; Rural-NA=Rural non-Adjacent; CI=Confidence Interval

[a]Estimates were derived from a logistic regression model. Covariates were: age, female sex, non-white race, Medicaid-eligibility, Elixhauser comorbidity index, hospital length of stay, admitting diagnosis, intensive care unit admission, and census region (Northeast, Midwest, South, and West).

© 2020 Kosar CM et al. *JAMA Network Open.*

eFigure 3. Adjusted[a] Rural-Urban Differences in Rates of Adverse Outcomes Among Individuals Admitted for Heart Failure

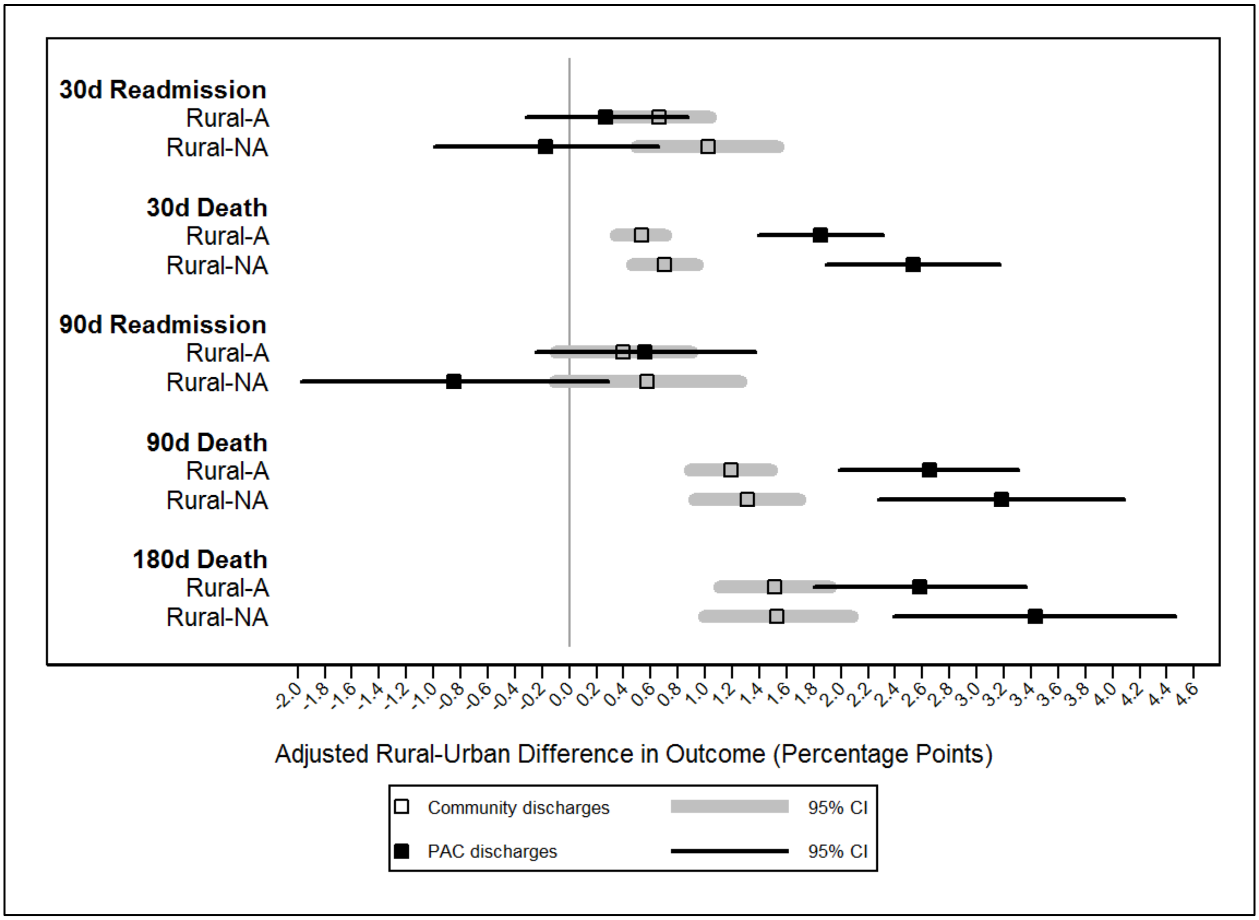

Abbreviations: PAC=Postacute care; Rural-A=Rural Adjacent; Rural-NA=Rural non-Adjacent; CI=Confidence Interval

[a]Estimates were derived from a logistic regression model. Covariates were: age, female sex, non-white race, Medicaid-eligibility, Elixhauser comorbidity index, hospital length of stay, admitting diagnosis, intensive care unit admission, and census region (Northeast, Midwest, South, and West).

© 2020 Kosar CM et al. *JAMA Network Open.*

eFigure 4. Adjusted[a] Rural-Urban Differences in Rates of Adverse Outcomes Among Individuals Admitted for Hip Fracture

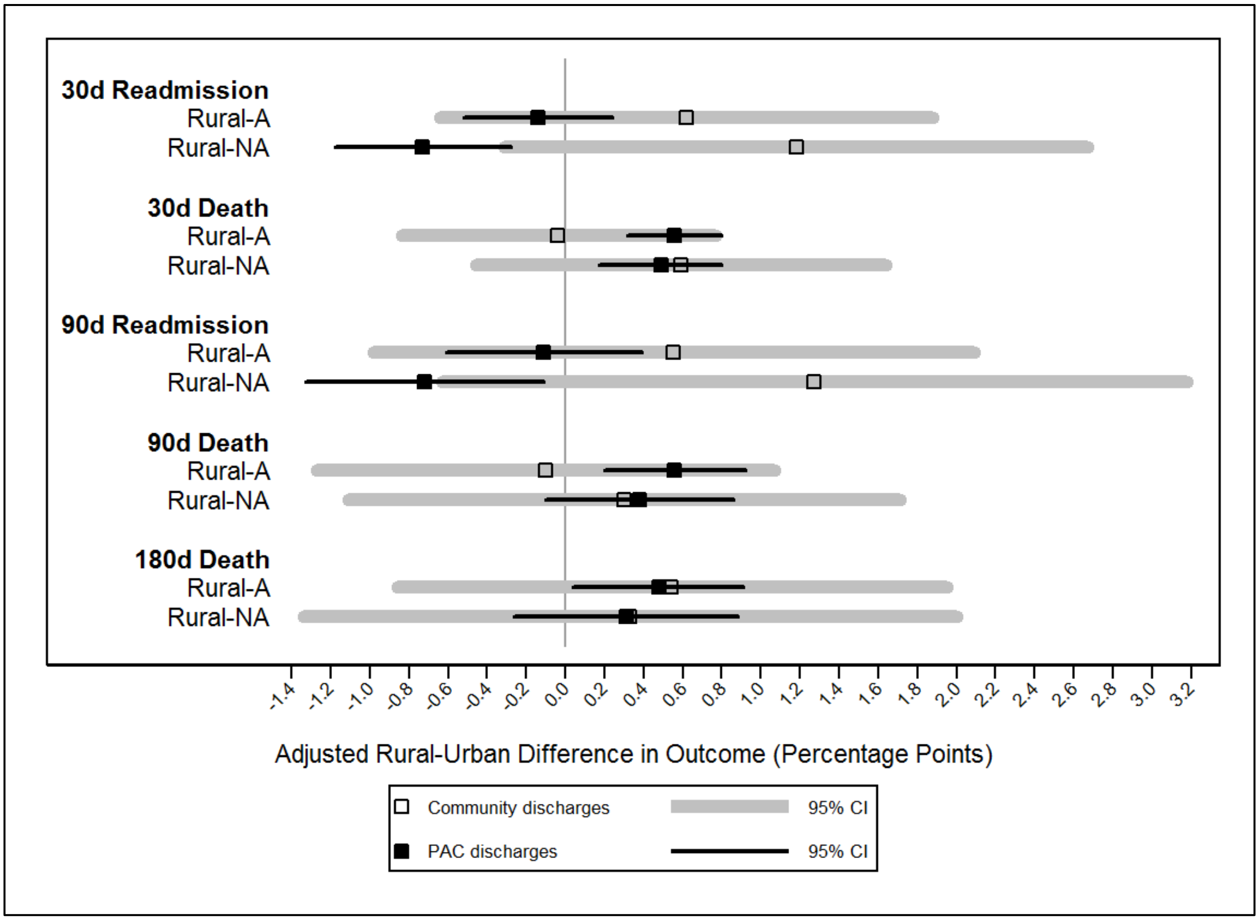

Abbreviations: PAC=Postacute care; Rural-A=Rural Adjacent; Rural-NA=Rural non-Adjacent; CI=Confidence Interval

[a]Estimates were derived from a logistic regression model. Covariates were: age, female sex, non-white race, Medicaid-eligibility, Elixhauser comorbidity index, hospital length of stay, admitting diagnosis, intensive care unit admission, and census region (Northeast, Midwest, South, and West).

© 2020 Kosar CM et al. *JAMA Network Open.*

eFigure 5. Adjusted[a] Rural-Urban Differences in Rates of Adverse Outcomes Among Individuals Admitted for Pneumonia

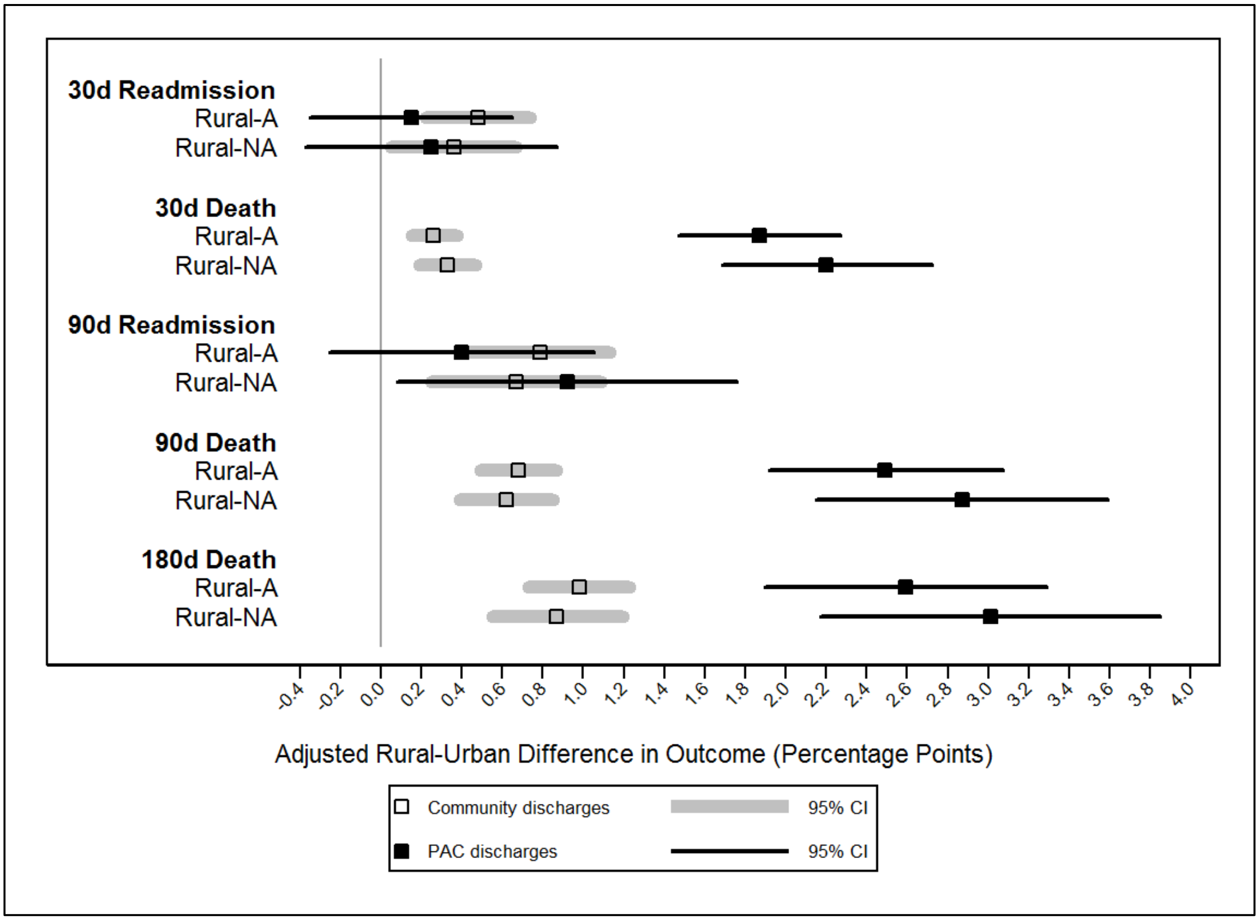

Abbreviations: PAC=Postacute care; Rural-A=Rural Adjacent; Rural-NA=Rural non-Adjacent; CI=Confidence Interval

[a]Estimates were derived from a logistic regression model. Covariates were: age, female sex, non-white race, Medicaid-eligibility, Elixhauser comorbidity index, hospital length of stay, admitting diagnosis, intensive care unit admission, and census region (Northeast, Midwest, South, and West).

© 2020 Kosar CM et al. *JAMA Network Open.*

eFigure 6. Rural-Urban Differences in 90-Day Health Care Utilization and Mortality Trajectories After Discharge to Institutional Postacute Care

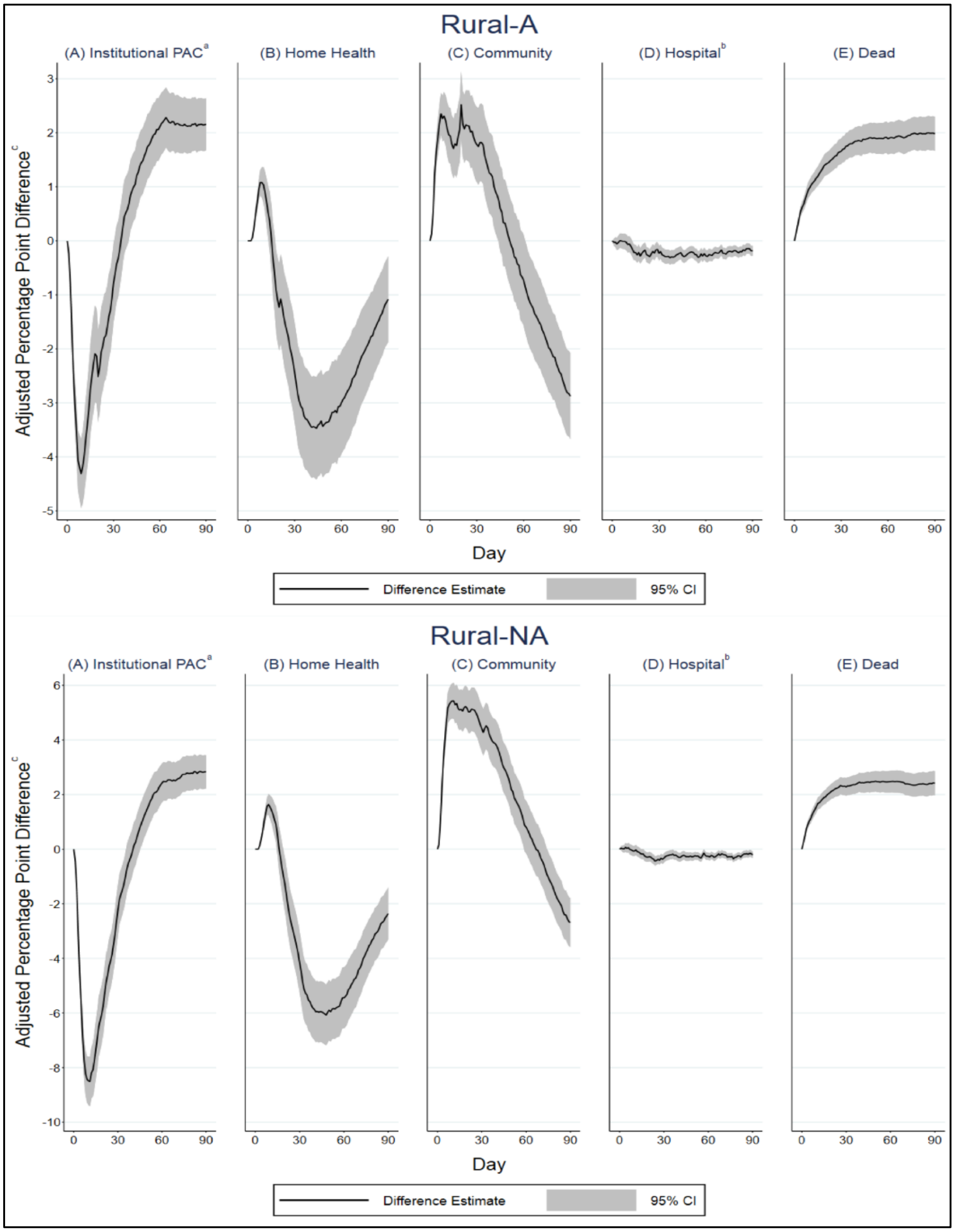

Abbreviations: PAC=Postacute care; Rural-A=Rural Adjacent; Rural-NA=Rural non-Adjacent; CI=Confidence Interval

[a]Individuals in institutional PAC (A) were either in a skilled nursing facility, nursing home, swing bed, or inpatient rehabilitation facility. [b]Hospital care (D) consists of individuals admitted to a general acute hospital, critical access hospital, or other inpatient setting (e.g. psychiatric hospital). [c]The adjusted proportion of individuals in each setting on each day were derived from multinomial logistic regression models adjusted for age, female sex, non-white race, Medicaid-eligibility, Elixhauser comorbidity index, hospital length of stay, admitting diagnosis, intensive care unit admission, and census region (Northeast, Midwest, South, and West).

© 2020 Kosar CM et al. *JAMA Network Open.*

eFigure 7. Rural-Urban Differences in 90-Day Health Care Utilization and Mortality Trajectories After Discharge to Home With Home Health

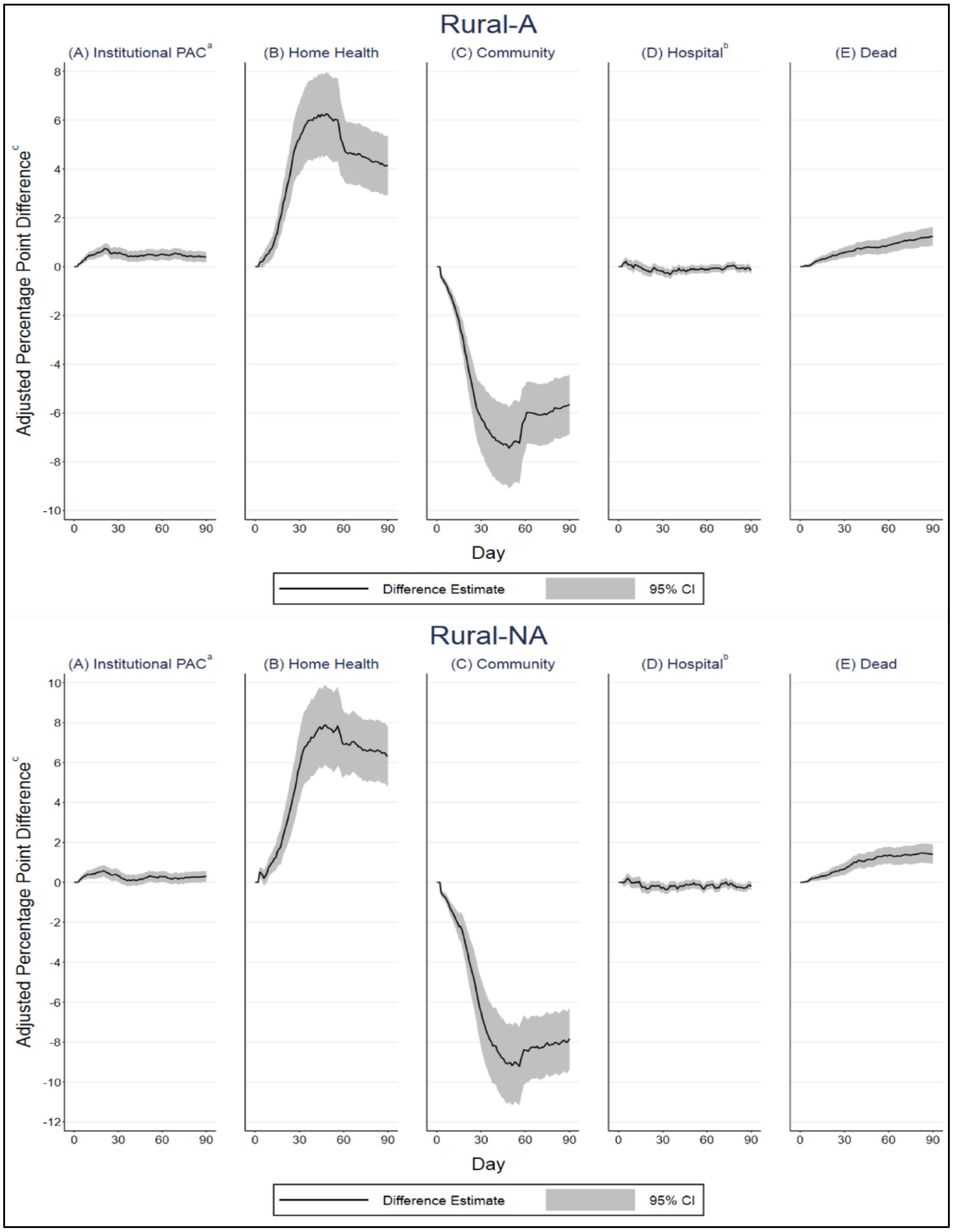

Abbreviations: PAC=Postacute care; Rural-A=Rural Adjacent; Rural-NA=Rural non-Adjacent; CI=Confidence Interval

[a]Individuals in institutional PAC (A) were either in a skilled nursing facility, nursing home, swing bed, or inpatient rehabilitation facility. [b]Hospital care (D) consists of individuals admitted to a general acute hospital, critical access hospital, or other inpatient setting (e.g. psychiatric hospital). [c]The adjusted proportion of individuals in each setting on each day were derived from multinomial logistic regression models adjusted for age, female sex, non-white race, Medicaid-eligibility, Elixhauser comorbidity index, hospital length of stay, admitting diagnosis, intensive care unit admission, and census region (Northeast, Midwest, South, and West).

© 2020 Kosar CM et al. *JAMA Network Open.*
